# Mutational profiling of brain metastasis from breast cancer: matched pair analysis of targeted sequencing between brain metastasis and primary breast cancer

**DOI:** 10.18632/oncotarget.6192

**Published:** 2015-10-20

**Authors:** Ji Yun Lee, Kyunghee Park, Sung Hee Lim, Hae Su Kim, Kwai Han Yoo, Ki Sun Jung, Haa-Na Song, Mineui Hong, In-Gu Do, TaeJin Ahn, Se Kyung Lee, Soo Youn Bae, Seok Won Kim, Jeong Eon Lee, Seok Jin Nam, Duk-Hwan Kim, Hae Hyun Jung, Ji-Yeon Kim, Jin Seok Ahn, Young-Hyuck Im, Yeon Hee Park

**Affiliations:** ^1^ Division of Hematology-Oncology, Department of Medicine, Samsung Medical Center, Sungkyunkwan University School of Medicine, Seoul, Korea; ^2^ Samsung Genomic Institute, Samsung Biological Research Institute, Samsung Medical Center, Sungkyunkwan University School of Medicine, Seoul, Korea; ^3^ Center of Companion Diagnostics, Innovative Cancer Medicine Institute, Samsung Medical Center, Sungkyunkwan University School of Medicine, Seoul, Korea; ^4^ Department of Surgery, Samsung Medical Center, Sungkyunkwan University School of Medicine, Seoul, Korea; ^5^ Department of Molecular Cell Biology, Samsung Biomedical Research Institute, Sungkyunkwan University School of Medicine, Suwon, Korea; ^6^ Biomedical Research Institute, Samsung Medical Center, Sungkyunkwan University School of Medicine, Seoul, Korea

**Keywords:** breast cancer, brain metastasis, gene, mutation, mechanism

## Abstract

Although breast cancer is the second most common cause of brain metastasis with a notable increase of incidence, genes that mediate breast cancer brain metastasis (BCBM) are not fully understood. To study the molecular nature of brain metastasis, we performed gene expression profiling of brain metastasis and matched primary breast cancer (BC). We used the Ion AmpliSeq Cancer Panel v2 covering 2,855 mutations from 50 cancer genes to analyze 18 primary BC and 42 BCBM including 15 matched pairs. The most common BCBM subtypes were triple-negative (42.9%) and basal-like (36.6%). In a total of 42 BCBM samples, 32 (76.2%) harbored at least one mutation (median 1, range 0–7 mutations). Frequently detected somatic mutations included TP53 (59.5%), MLH1 (14.3%), PIK3CA (14.3%), and KIT (7.1%). We compared BCBM with patient-matched primary BC specimens. There were no significant differences in mutation profiles between the two groups. Notably, gene expression in BCBM such as TP53, PIK3CA, KIT, MLH1, and RB1 also seemed to be present in primary breast cancers. The TP53 mutation frequency was higher in BCBM than in primary BC (59.5% vs 38.9%, respectively). In conclusion, we found actionable gene alterations in BCBM that were maintained in primary BC. Further studies with functional testing and a delineation of the role of these genes in specific steps of the metastatic process should lead to a better understanding of the biology of metastasis and its susceptibility to treatment.

## INTRODUCTION

Breast cancer (BC) is the second-most common cancer that spreads to the brain [1]. The prevalence of breast cancer brain metastasis (BCBM) has been reported to range from 10–16%, reaching 30% when autopsy diagnoses of brain metastasis are included [1, 2]. The median survival after development of BCBM is approximately 4–5 months [3]. Breast cancer patients with triple-negative (TN), basal-like, HER2-positive tumors are at the highest risk of brain cancer relapse [4–6]. However, the molecular basis of mechanisms responsible for BM remains elusive because the brain is a special challenge for tumor cells due to the blood-brain barrier (BBB).

Organ-specific metastasis has been associated with a set of genes that are involved in metastatic processes such as tumor cell intravasation, survival in circulation, extravasation into a distant organ, angiogenesis and uninhibited growth [7, 8]. Most research regarding BCBM development has been based on gene expression profiling of BC coupled with clinical data, functional analysis on cell lines, and *in vivo* animal models [9–11]. Recently, there have been many studies on the gene expression profile of BCBM compared to their matched primary BC. Silva et al. suggests that increased activation of *HER3* and its downstream MAPK/AKT pathway molecules are implicated in colonization of brain metastasis [12]. Bolling-Fischer et al. showed the amplified oncogenes including *SOX2, PIK3CA, NTRK1, GNAS, CTNNB1*, and *FGFR1* are related to the Stem Cell Pluripotency pathway [13]. Saunus et al. identified novel candidates with possible roles in BCBM development including the significantly mutated genes *DSC2, ST7, PIK3R1*, and *SMC5* [14]. However, the clinical relevance of many existing candidates is not fully understood. Therefore, we aim to identify genes that are correlated with the propensity of primary BC to brain cancer relapse using matched tissue samples from BCBM and primary BC.

## RESULTS

### Patient characteristics

Patient demographics are summarized in Table 1. Median age at diagnosis of BC was 45 years. The majority of patients were premenopausal woman (79.5%) and the most common histology was invasive ductal carcinoma (88.1%). Five (11.9%) patients were initially diagnosed as stage IV metastatic disease. Among 45 patients, the proportion of ER+, ER+/HER2+, HER2+, and TNBC in breast cancer tissue was 31.7%, 9.8%, 26.8%, and 31.7%, respectively. The median time to brain metastasis from curative resection and median overall survival from BCBM was 2.5 years (range, 0–17.7 years) and 1.9 years (range, 0.3–6.7 years), respectively. Among the 42 BCBM samples, the distribution by tumor subtype according to the immunohistochemistry (IHC) included 42.9% TN, 26.2% ER+, 19.0% HER2+, and 11.9% ER+/HER2+ type (Table 1). In the same group, PAM50 subtypes included 36.6% basal-like, 31.7% Her2-enriched, 29.3% luminal (A or B), and 2.4% normal-like type (Table 1).

**Table 1:** Baseline characteristics

			*N* = 45	%
Median age (range), years			44.6 (22.4–64.1)	
Menopausal status				
Premenopausal			31	79.5
Postmenopausal			8	20.5
Histology				
Invasive ductal carcinoma			37	88.1
Invasive lobular carcinoma			1	2.4
Others			4	9.5
Grade				
Low			0	0
Intermediate			11	34.4
High			21	65.6
Stage				
I			7	16.7
II			17	40.5
III			13	31.0
IV			5	11.9
Median time to brain metastasis (range)^*^, years			2.5 (0–17.7)	
Median overall survival (range)^†^, years			5.1 (0.8–20.0)	
**Subtype**	**Breast (*N* = 18)**		**Brain (*N* = 42)**	
	***N***	**%**	***N***	**%**
IHC				
ER+	5	27.8	11	26.2
ER+/HER2+	0	0	5	11.9
HER2+	6	33.3	8	19.0
TN	7	38.9	18	42.9
PAM50				
Luminal A	6	33.3	4	9.8
Luminal B	0	0	8	19.5
Her2-enriched	6	33.3	13	31.7
Basal-like	5	27.8	15	36.6
Normal-like	1	5.6	1	2.4

* median time to brain metastasis from curative resection

† median overall survival from brain metastasis

### Mutation analysis using the Iron AmpliSeq cancer panel (MAF > 0.1)

**Figure 1:**
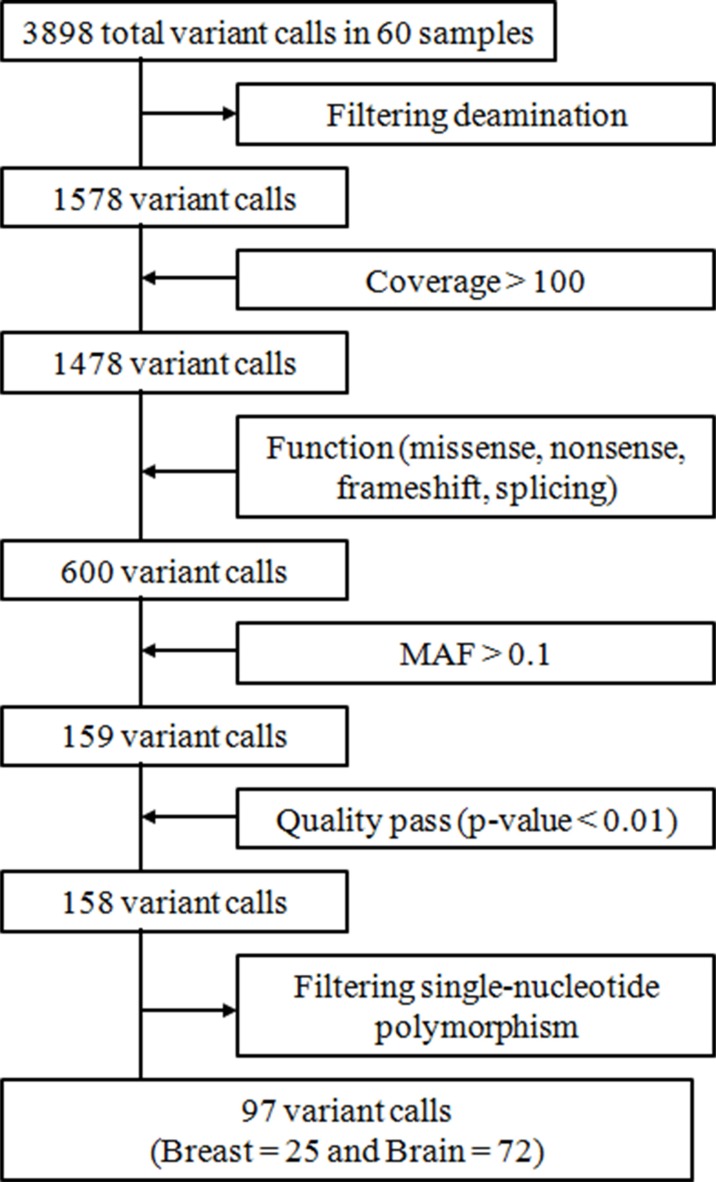
Summary of variant call processing

To identify patterns of gene expression associated with BCBM, we performed a NGS using the Iron AmpliSeq cancer panel. In total, we obtained 3898 variant calls from 60 samples and 97 variant calls were selected: 25 variant calls with 23 mutations for primary BC and 72 variant calls with 64 mutations for BCBM (Figure 1). The most common genetic alterations were somatic single nucleotide variants (SNVs) (82/97, 84.5%) and small insertion-deletions (indels) (12/97, 12.4%). Although there were no significant differences in mutation type according to the tumor tissue, proportion of SNVs was numerically higher in primary BC group (96.0% *vs* 80.6%, *P* = 0.187). Detailed frequency of mutations and amino acid changes in 60 samples are described in Table S2.

**Figure 2:**
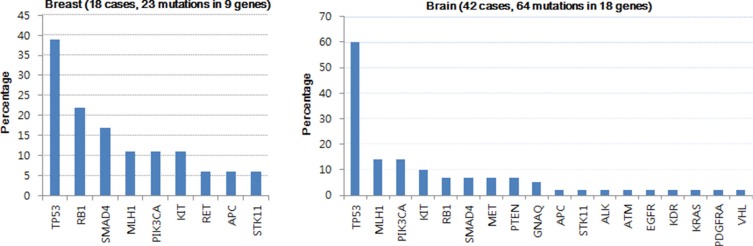
Frequency of mutations in 60 patients for Ampliseq (MAF > 0.1) **(a)** primary breast cancer and **(b)** brain metastasis from the breast.

Figure 2 shows the frequency of mutations in 50 genes among 60 patients according to the tissue origin. The frequency of mutations was not significantly different between primary BC and BCBM (*P* = 0.475). When using the 50-cancer gene panel in 18 primary BC samples, 14 of 18 patients (77.8%) had at least one mutation (median 1, range 0–4 mutations). Among the 23 mutations in primary BC, the frequency of mutations according to subtype was as follows: TN (43.5%), ER+ (34.8%), HER2+ (21.7%), and ER+/HER2+ (0%) for IHC and luminal A (39.1%), HER2-enriched (34.8%), and basal-like (26.1%) for PAM50 (Table S3). Among the 18 primary BC cases, the most common mutations included *TP53* (7, 38.9%), *RB1* (4, 22.2%), *SMAD4* (3, 16.7%), *MLH1* (2, 11.1%), *PIK3CA* (2, 11.1%), and *KIT* (2, 11.1%).

**Figure 3:**
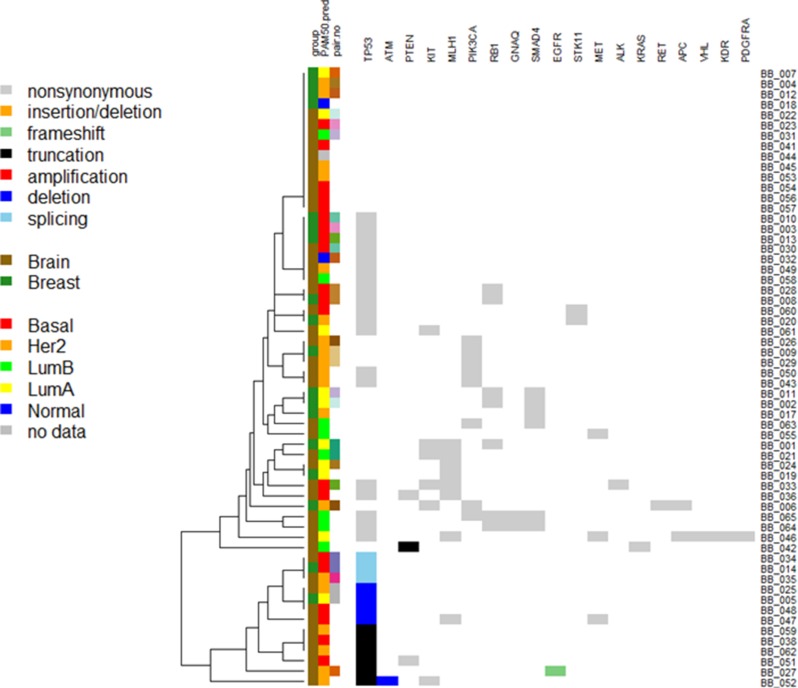
Heatmap of the mutations found in 60 patients

In a total of 42 BCBM samples, 32 (76.2%) harbored at least one mutation (median 1, range 0–7 mutations). Among the 64 mutations in BCBM, the frequency of mutation according to the subtypes was as follows: TN (39.1%), ER+ (32.8%), HER2+ (17.2%), and ER+/HER2+ (10.9%) for IHC and basal-like (31.3%), luminal B (26.6%), HER2-enriched (25.0%), luminal A (15.6%) and normal-like (1.5%) for PAM50 (Table S3). Among the 42 BM cases, *TP53* was the most common mutation (25, 59.5%). Other mutations included *MLH1* (6, 14.3%), *PIK3CA* (6, 14.3%), and *KIT* (3, 7.1%). Figure 3 depicts the heat map of the mutations detected in the 60 samples.

Among the 30 *TP53* mutations detected in BCBM, 25 (83.3%) occurred in exons 5–8, which is the DNA binding domain (Table S4). The majority of *TP53* mutations were missense mutations (15, 50.0%). Other alterations included frameshift insertion/deletions (8, 26.7%), nonsense mutations (4, 13.3%), splicing (2, 6.7%), and inframe insertion/deletions (1, 3.3%). Frameshift, splicing, and nonsense mutations and in-frame insertions and deletions constitute complex *TP53* mutations [15]. In terms of the PAM50 subtype, complex mutations were observed in HER2-enriched (8/15, 53.3%) and basal-like (7/15, 46.7%).

### Comparison between breast cancer and brain metastasis

**Figure 4:**
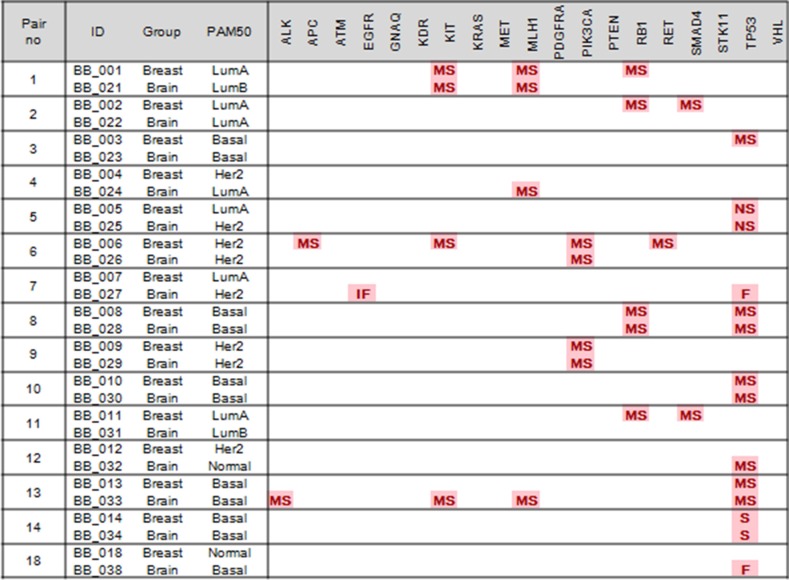
Gene expression profiles of the primary breast cancer were compared to those of brain metastasis from breast cancer (MS, missense; NS, non-sense; F, frameshift; IF, in-frame insertion/deletion; S, splicing)

Tumor progression is considered the result of cumulative oncogenic alterations. We hypothesized that tissue-specific genes for metastasis are superimposed on the breast gene expression signature. We compared mutation profiles of primary BC (*N* = 18) and BCBM (*N* = 42) in order to identify gene expression signatures associated with BM. There was no significant difference in mutation profiles between the two groups (Table 2). *RB1* mutations were found in 3 (7.1%) and 4 cases (22.2%) in the BM and BC groups, respectively (*P* = 0.182). Next, we explored the patient-matched pair samples with primary BC and BCBM. Discordant expression of PAM50 molecular subtypes and IHC was observed between primary BC and BCBM (Table S5). A PAM50 molecular subtype conversion was observed in 7/15 (46.7%). In IHC, 2/15 (13.3%) paired cases had discordant ER expression, all of which were loss of ER. Genetic alterations such as *TP53, PIK3CA, KIT, MLH1*, and *RB1* were detected in both primary BC and BCBM in the same patients (Figure 4). *RB1* mutations were observed more frequently in primary BC samples than in BCBM samples (26.7% *vs* 7.1%, *P* = 0.330). *SMAD4* mutations were identified in 2 cases (13.3%) among 15 primary BC samples, but no mutation was observed in BCBM samples. In all 15 pairs, 5 pairs including #5, #8, #9, #10 and #14 had identical genetic alterations. Three paired sets (#1, #6, and #13) demonstrated a set of conserved cancer gene aberrations, though two sets showed additional cancer gene lesions in the primary BC and the other had gains in the BCBM. In pair #6, 4 mutations including *APC*, *KIT, PIK3CA*, and *RET* were detected in the primary lesion, but only the *PIK3CA* mutation was detected in the BCBM. On the other hand, in pair #13, only a *TP53* mutation was observed in primary BC, but 4 mutations, *ALK, KIT, MLH1*, and *TP53*, were observed in the BCBM.

**Table 2:** Comparison of mutation profiles using Ampliseq (MAF > 0.1)

Data set	Group 1 *vs* Group 2	Gene	Group 1 wild	Group 1 mut	Group 2 wild	Group 2 mut	*P* value	Group 1 ratio	Group 2 ratio
All (*N* = 60)	Brain *vs* Breast	TP53	17	25	11	7	0.167	0.5952	0.3889
		RB1	39	3	14	4	0.182	0.0714	0.2222
Pair (*N* = 15)	Brain *vs* Breast	RB1	14	1	11	4	0.330	0.0667	0.2667
		SMAD4	15	0	13	2	0.483	0	0.1333

### Analysis of paired primary breast and brain metastasis

Next, we aimed to identify candidate *TP53* mutations with possible roles in BC development. To explore the molecular differences of *TP53* between primary BC and BCBM, we compared the *TP53* gene expression profiles of BCBM to matched primary BC (Table 3). In all 9 pairs, 4 pairs including #5, #8, #10, and #14 had identical *TP53* mutations. In pair #3, His179Tyr was observed in breast cancer, but no His179Tyr mutation was observed in brain metastasis. In 3 pairs, the *TP53* mutation was detected in BCBM, but no mutation was observed in the primary BC: Met340Thr (frameshift insertion) for #7, Leu257Arg (missense) for #12, and Arg342Glu (frameshift deletion) for #18.

**Table 3:** TP53 mutations in brain metastasis with matched primary breast cancer (9 pairs, 8 mutations in bain; 6 mutations in breast)

Pair No.	Case No.	Tissue	IHC	PAM50	coding	Function	Protein
3	BB_003	Breast	TN	Basal	c.535C > T	[missense]	p.His179Tyr
	BB_023	Brain	TN	Basal			
5	BB_005	Breast	ER+	LumA	c.497C > G	[nonsense]	p.Ser166*
	BB_025	Brain	ER+	Her2	c.497C > G	[nonsense]	p.Ser166*
7	BB_007	Breast	ER+	LumA			
	BB_027	Brain	TN	Her2	c.1013_1014insCGAGA	[frameshiftInsertion]	p.Met340Thr
8	BB_008	Breast	TN	Basal	c.584T > C	[missense]	p.Ile195Thr
	BB_028	Brain	TN	Basal	c.584T > C	[missense]	p.Ile195Thr
10	BB_010	Breast	TN	Basal	c.838A > G	[missense]	p.Arg280Gly
	BB_030	Brain	TN	Basal	c.838A > G	[missense]	p.Arg280Gly
12	BB_012	Breast	HER2+	Her2			
	BB_032	Brain	HER2+	Normal	c.770T > G	[missense]	p.Leu257Arg
13	BB_013	Breast	TN	Basal	c.329G > C	[missense]	p.Arg110Pro
	BB_033	brain	TN	Basal	c.535C > T	[missense]	p.His179Tyr
14	BB_014	Breast	TN	Basal	chr17:7577610T > C	splicing	
	BB_034	Brain	TN	Basal	chr17:7577610T > C	splicing	
18	BB_018	Breast	TN	Normal			
	BB_038	brain	TN	Basal	c.1024_1024delC	[frameshiftDeletion]	p.Arg342Glu

## DISCUSSION

Current therapeutic strategies for BCBM include whole brain radiation therapy, stereotactic radiosurgery, and surgery combined with radiotherapy [16, 17]. Recently, systemic treatments such as chemotherapy and targeted therapy after local therapy improved survival in patients with BCBM [18, 19]. Nevertheless, BCBM has limited life expectancy since chemotherapeutic and targeted agents penetrate the BBB. In the whole genome sequencing era, genomic and transcriptomic analysis may uncover new drug agents targeting BCBM. However, studies of genetic alterations in BCBM have been limited by the lack of tissue availability. To the best our knowledge, this is the largest study to demonstrate the gene expression profiles of brain metastasis and matched primary breast cancer using targeted sequencing.

We performed genome-wide aberration profiling on 18 primary BC and 42 BCBM tissue samples and compared the properties of mutations between these two tumor groups. The set of BCBM samples was enriched for TN/basal-like BC, which is consistent with previous reports of an increased propensity of metastasis to the brain [5, 20]. Furthermore, the frequency of genetic alterations was closely linked to subtype in BCBM. We found that 76.2% of BCBM patients harbored at least one mutation. Known mutated drivers of primary breast were frequently mutated in BCBM including *TP53, MLH1, PIK3CA*, and *KIT*. When we analyzed matched pairs of primary BC and BCBM, genetic alterations such as *TP53, PIK3CA, KIT, MLH1*, and *RB1* were detected in both primary BC and BCBM in the same patients. Besides our data, other recent findings revealed the existence of metastasis gene signatures expressed by primary tumors [21–23]. Based on these studies, those mutations occurred in the primary carcinoma, and then some of the cell population metastasized to the brain. However, those mutations show that the cells have accumulated a sufficient number of malignant functions to promote expansion of the primary tumor, but not sufficient for forming metastasis [24]. Recent studies suggest that distant metastasis occurs late during the genetic evolution of primary cancer [25, 26]. We anticipated that if there were discordances between primary BC and BCBM, genetic alterations could be enriched in BCBM compared to primary BC. Unexpectedly, the RB1 and SMAD4 mutations were observed more frequently in primary BC samples, although it should be cautious to make conclusion due to limited number of study population. The tumor suppressor RB1 is often lost by mutation, deletion or transcriptional silencing in may human malignancies [27–29]. RB1 is primarily inactivated in TNBC (∼20%) [30]. Gupa et al. showed that RB1 loss was associated with unfavorable distant metastasis-free survival in TNBC [31]. The functional loss of RB1 may play a key role in aggressive biology, but its role in metastatic process is unknown. Smad4 is a central mediator of transforming growth factor-Δ (TGF-Δ) intracellular signaling [32]. Smad 4 mutation is most prevalent in pancreatic and colorectal cancer [32]. Many studies showed that Smad4 alterations were more frequent in advanced cancer and in metastatic cancers [33, 34]. However, little is known about the expression level of Smad4 or its prognostic significance in breast cancer. Deckers et al. demonstrated that TGF-Δ-induced growth inhibition and apoptosis, TGF-Δ-induced EMT, and metastasis of breast cancer cells to bone were critically dependent on Smad4 [35]. Fewer frequencies of SMAD4 mutation in BCBM may be attributed that SMAD4 plays a dual role in carcinogenesis, being a tumor suppressor and a tumor promoter in different stages, although further investigations are necessary to confirm these findings.

The p53 tumor suppressor plays a critical role in many cellular pathways controlling cell proliferation, cell survival, and genomic integrity [36]. In breast cancer, the *TP53* mutation is associated with more aggressive disease and worse overall survival [37, 38]. Tham et al. showed that *TP53* alterations predicted BCBM [39]. However, the contribution of *TP53* to BM is poorly understood. According to the various studies, mutations in *TP53* occur in 20% of primary BC and the majority of *TP53* mutations are missense substitution (75%) [37, 38]. In the current study, BCBM has an increased frequency of *TP53* mutations with distinct properties compared with those found in primary BC. We found a higher frequency of *TP53* mutations in BCBM (59.5% *vs.* 38.9%), although it was not statistically significant. In addition, the frequency of complex *TP53* mutations was up to 50% in BCBM. Consistent with our study, a recent study reported by Nigro et al. demonstrated a high frequency (87%) of *TP53* mutations with an over-representation of complex mutations (45%) [40]. The frequency of complex mutations is reported to be higher in the basal-like subtype of BC [37]. Indeed, the increased frequency of complex *TP53* mutations in brain metastasis might be caused by an increased in basal-like type in BCBM over primary BC (36.6% *vs.* 27.8%). p53 directly influences transcription of genes involved in metastasis by binding to the promoters of various genes known to be involved in regulating cell motility and adhesion, processes that are important for metastasis [41, 42]. Genetic alteration of *TP53* not only aids in tumor initiation and progression, but also allows tumors to acquire metastatic facilitators that may suggest that *TP53* mutations are a prerequisite for the development of BCBM. A structural and functional analysis of *TP53* mutations is needed to develop a comprehensive understanding of BCBM.

The present study has several limitations. Although we applied multiple filters to prioritize genes and pathways of interest, the interpretation of the results should be cautious given the retrospective nature and small sample size of the current study. In addition, variables such as protein-protein interactions, transcriptional repression, and transactivation of other genes should be taken into account for an understanding of BCBM mechanisms. The specific organ microenvironment determines the extent of cancer cell proliferation, angiogenesis, invasion and survival [43]. Histological analysis of resected human brain metastasis revealed tumor cells interdigitated with activated microglia and astrocytes [44–46]. Considering that the organ microenvironment can influence the biology of metastasis, further study on the interactions between tumor cells and the host environment is needed.

In conclusion, we explored paired analysis of mutational profiling between primary BC and BCBM. Major gene mutations may have a role in metastasis to the brain from BC, though it we could not show any difference between primary BC and BCBM.

## MATERIALS AND METHODS

### Patients

All samples were collected from breast cancer patients who underwent surgical resection at the Samsung Medical Center. The cohort consisted of samples from 18 BC and 42 BM patients. Fifteen matched pairs of primary BC and BCBM samples were available. All patients provided written informed consent. This study was performed in accordance with the Declaration of Helsinki and approved by Institutional Review Board of Samsung Medical Center (SMC 2013–12–155).

### Immunohistochemistry

Two experienced pathologists reviewed all pathology specimens to determine the following tumor characteristics: histological and nuclear grades, primary tumor size, presence of lymphovascular invasion, multiplicity, and IHC staining for ER, PgR, and HER2. ER and PgR positivity were defined using Allred scores ranging from 3 to 8 based on IHC using antibodies to the ER (Immunotech, Marseille, France) and PgR (Novocastra Laboratories Ltd., Newcastle upon Tyne, UK). HER2 status was evaluated using a specific antibody (Dako, Glostrop, Denmark) and/or fluorescence *in situ* hybridization (FISH). Grades 0 and 1 for HER2, as assessed by IHC, were defined as a negative result, and grade 3 was defined as a positive result. Amplification of HER2 was confirmed by FISH if HER2 was rated as 2 + by IHC.

### DNA extraction/RNA extraction

A total of 60 tissue samples including 18 primary BCs and 42 BCBMs from 45 patients with a tumor cell percentage of more than 75% (from 4-mm unstained sections) were dissected under a microscope by comparison to an H&E-stained slide. Genomic DNA was extracted using the Qiagen DNA FFPE Tissue Kit (Qiagen, Hilden, Germany) and total RNA was extracted using the High Pure RNA Paraffin kit (Roche Diagnostics, Mannheim, Germany), according to the manufacturer's instructions. After extraction, we measured DNA and RNA concentration using a spectrophotometer (ND1000; NanoDrop Technologies, Thermo Fisher Scientific, Waltham, MA, USA). Each sample was then quantified using a Qubit fluorometer (Life Technologies, Carlsbad, CA, USA). Samples with less than 10 ng/μL of genomic DNA and less than 50 ng/uL of total RNA, even after concentration using a SpeedVac concentrator (Thermo Scientific™, Waltham, MA, USA) were excluded from downstream analysis.

### Next-generation sequencing (NGS) using Ion torrent ampliseq cancer panel v2

Using the Ion Torrent Personal Genome Machine (Ion PGM, Life Technologies, Carlsbad, CA, USA) Cancer Panel v2 (Table S1) after DNA isolation from formalin-fixed, paraffin-embedded (FFPE) samples, we sequenced 2,855 loci from 50 cancer-related genes to identify genetic mutations in 60 samples from BC patients. Libraries were constructed using the Ion AmpliSeq Panels pool (Life Technologies) with a 10-ng DNA sample per pool. The amplicons were then ligated to Ion Xpress Barcode Adapters and purified. Next, multiplexed bar-coded libraries were enriched by clonal amplification using emulsion PCR on Ion Sphere particles (Ion PGM Template OT2 200 Kit, Life Technologies) and loaded onto an Ion 316 Chip. Massively parallel sequencing was carried out on the Ion PGM using the Ion PGM Sequencing 200 Kit v2. The Ion AmpliSeq Cancer Hotspot Panel v2 (www.lifetechnologies.com) covered hot-spot regions of 50 oncogenes and tumor suppressor genes.

The primary filtering process was carried out using the Torrent Suite v3.6.0 and the Ion Torrent Variant Caller v3.6 software. The pipeline includes signaling processing, base calling, quality score assignment, adapter trimming, read alignment to human genome 19 references, mapping QC, coverage analysis, and variant calling. For variant detection, a minimum coverage of 100 reads must be achieved, and at least 5% of mutant reads were selected for variants. Variant calls were further analyzed using the ANNOVAR, which included variant filtering and annotation using the COSMIC database, dbSNP build 137, and amino acid change information.

### Sample subtype prediction

PAM50 genes expression was measured on the NanoString nCounter Analysis System (NanoString Technologies, Seattle, WA, USA). The system measures the relative abundance of each mRNA transcript of interest using a multiplexed hybridization assay and digital readouts of fluorescent bar-coded probes that are hybridized to each transcript are created [47]. Intrinsic subtype classification was performed using the PAM50 predictor and was applied to the nearest PAM50 centroid algorithm Bioclassifier to predict the PAM50 subtypes, as described in Parker et al. [48]. To obtain more consistent results, we merged microarray expression data of TCGA breast cancers with our NanoString data after adjusting for batch effects using the ComBat algorithm [49] and applied the nearest PAM50 centroid algorithm Bioclassifier to predict PAM50 subtypes [48]. For all statistical tests, PAM50 subtype prediction was conducted using R version 3.0.2 (http://www.R-project.org/).

### Bioinformatic and statistical analysis for ampliseq

To obtain results more accurately, the final data were filtered through several steps. In the first step, samples with cytosine deamination were removed. In the second step, false positive site were removed under the following conditions; the coverage (> 100X) and *P*-value < 0.01. In addition, a minimum threshold of mutant allele fraction (MAF) was taken into account to determine if the variant was real: > 10% for mutations with a low allele fraction. For the statistical analysis of final variants, read alignments were manually investigated using the Integrative Genomic Viewer (http://www.broadinstitute.org/igv/). We filtered out single-nucleotide polymorphisms after manual review of each polymorphism in the Catalogue of Somatic Mutations in Cancer (COSMIC, http://cancer.sanger.ac.uk/ cancergenome/projects/cosmic). We also discarded the Korean-specific germ-line variants rs1042522 in TP53 and rs1870377 in KDR. Among the variants that satisfied the filtering criteria described above, variants causing amino acid changes and frameshifts were finally chosen for statistical analysis. Fisher's exact test was used for the analysis of mutations and polymorphic variants separately, to discover variants that were enriched in patients with favorable outcomes. *P*-values < 0.05 were considered significantly different.

## SUPPLEMENTARY MATERAL TABLES




