# Supplementary material for: Mutational profiling of brain metastasis from breast cancer: matched pair analysis of targeted sequencing between brain metastasis and primary breast cancer
Source: Oncotarget. 2015 Oct 20;6(41):43731–42. doi: 10.18632/oncotarget.6192 (PMC4791262; doi:10.18632/oncotarget.6192)
Supplement: Supplementary file 1 [file oncotarget-06-43731-s001.pdf]

Mutational profiling of brain metastasis from breast cancer: matched pair analysis of targeted sequencing between brain metastasis and primary breast cancer

Supplementary Materials

Supplementary Table 3: Frequency of mutation according to the subtype of breast cancer

			Variant calls (*N* = 25)		Mutation (*N* = 23)	
			N	%	*N*	%
Breast	IHC	ER+	8	32.0	8	34.8
		ER+/HER2+	0	0	0	0
		HER2+	5	20.0	5	21.7
		TN	12	48.0	10	43.5
	PAM50	LumA	9	36.0	9	39.1
		LumB	0	0	0	0
		HER2	9	36.0	8	34.8
		Basal	7	28.0	6	26.1
		Normal	0	0	0	0
			Variant calls (*N* = 72)		Mutation (*N* = 64)	
			N	%	*N*	%
Brain	IHC	ER+	24	33.3	21	32.8
		ER+/HER2+	7	9.7	7	10.9
		HER2+	12	16.7	11	17.2
		TN	29	40.3	25	39.1
	PAM50	LumA	13	18.1	10	15.6
		LumB	17	23.6	17	26.6
		HER2	19	26.4	16	25.0
		Basal	22	30.5	20	31.3
		Normal	1	1.4	1	1.5

Supplementary Table 4: TP53 mutations in brain metastasis

Case No.	IHC	PAM50	Exon	Coding	Function	Protein
BB_025	ER+	Her2	7	c.497C > G	[nonsense]	p.Ser166*
BB_027	TN	Her2	2	c.1013_1014insCGAGA	[frameshiftInsertion]	p.Met340Thr
BB_028	TN	Basal	6	c.584T > C	[missense]	p.Ile195Thr
BB_030	TN	Basal	8	c.838A > G	[missense]	p.Arg280Gly
BB_032	HER2+	Normal	5	c.770T > G	[missense]	p.Leu257Arg
BB_033	TN	Basal	7	c.535C > T	[missense]	p.His179Tyr
BB_034	TN	Basal	5	chr17:7577610T > C	[splicing]	
BB_035	ER+/ HER2+	Her2	7	chr17:7578555C > A	[splicing]	
BB_036	TN	Basal	7	c.536A > G	[missense]	p.His179Arg
BB_038	TN	Basal	2	c.1024_1024delC	[frameshiftDeletion]	p.Arg342Glu
BB_043	TN	Her2	5	c.740_745delACCGGA	[nonframeshiftDeletion]	p.Asn247Arg
			5	c.737T > A	[missense]	p.Met246Lys
BB_046	ER+	LumA	6	c.596G > A	[missense]	p.Gly199Glu
BB_047	TN	Basal	6	c.637C > T	[nonsense]	p.Arg213*
BB_048	TN	Basal	8	c.321C > G	[nonsense]	p.Tyr107*
BB_049	HER2+	Her2	6	c.584T > A	[missense]	p.Ile195Asn
BB_050	HER2+	Her2	7	c.392A > T	[missense]	p.Asn131Ile
BB_051	ER+	Basal	6	c.585_586insC	[frameshiftInsertion]	p.Arg196Pro
BB_052	HER2+	Her2	8	c.216_216delC	[frameshiftDeletion]	p.Val73Trp
BB_058	ER+/ HER2+	LumB	7	c.488A > G	[missense]	p.Tyr163Cys
BB_059	HER2+	Her2	4	c.846_846delG	[frameshiftDeletion]	p.Arg283Ala
BB_060	TN	Basal	6	c.637_637delC	[frameshiftDeletion]	p.Arg213Asp
			6	c.637C > T	[nonsense]	p.Arg213*
			10	c.31G > C	[missense]	p.Glu11Gln
BB_061	ER+	LumA	5	c.743G > A	[missense]	p.Arg248Gln
			10	c.31G > C	[missense]	p.Glu11Gln
BB_062	HER2+	Her2	6	c.626_627delGA	[frameshiftDeletion]	p.Arg209Lys
			6	c.626_626delG	[frameshiftDeletion]	p.Arg209Lys
BB_064	ER+	LumB	5	c.725G > T	[missense]	p.Cys242Phe
BB_065	ER+/ HER2+	LumB	7	c.476C > T	[missense]	p.Ala159Val

Supplementary Table 5: PAM 50 molecular subtype and immunohistochemistry in 15 paired cases

Case No.	Pair No.	Group	PAM50	IHC
BB_001	1	Breast	LumA	ER+
BB_021	1	Brain	LumB	TN
BB_002	2	Breast	LumA	ER+
BB_022	2	Brain	LumA	ER+
BB_003	3	Breast	Basal	TN
BB_023	3	Brain	Basal	TN
BB_004	4	Breast	Her2	HER2+
BB_024	4	Brain	LumA	HER2+
BB_005	5	Breast	LumA	ER+
BB_025	5	Brain	Her2	ER+
BB_006	6	Breast	Her2	TN
BB_026	6	Brain	Her2	TN
BB_007	7	Breast	LumA	ER+
BB_027	7	Brain	Her2	TN
BB_008	8	Breast	Basal	TN
BB_028	8	Brain	Basal	TN
BB_009	9	Breast	Her2	HER2+
BB_029	9	Brain	Her2	HER2+
BB_010	10	Breast	Basal	TN
BB_030	10	Brain	Basal	TN
BB_011	11	Breast	LumA	ER+
BB_031	11	Brain	LumB	ER+
BB_012	12	Breast	Her2	HER2+
BB_032	12	Brain	Normal	HER2+
BB_013	13	Breast	Basal	TN
BB_033	13	Brain	Basal	TN
BB_014	14	Breast	Basal	TN
BB_034	14	Brain	Basal	TN
BB_018	18	Breast	Normal	TN
BB_038	18	Brain	Basal	TN
